# A population study on factors associated with unintentional falls among Iranian older adults

**DOI:** 10.1186/s12877-023-04571-0

**Published:** 2023-12-15

**Authors:** Gholam Reza Sotoudeh, Reza Mohammadi, Zahra Mosallanezhad, Eija Viitasara, Joaquim J.F. Soares

**Affiliations:** 1 Department of Health Sciences, Section of Public Health Sciences, Mid Sweden University, Sundsvall, Sweden; 2 Sina Trauma and Surgery Research Center, Tehran University of Medical Sciences, Tehran, Iran; 3 Division for Family Medicine, Department of Neurobiology, Care Sciences and Society, Karolinska Institutet, Stockholm, Sweden; 4 Department of Physiotherapy, University of Social Welfare and Rehabilitation Sciences, Tehran, Iran; 5 Research Center on Aging, University of Social Welfare and Rehabilitation Sciences, Tehran, Iran; 6 Faculdade de Ciências Sociais e Tecnologia da Universidade Europeia, Universidade Europeia, Lisboa, Portugal

**Keywords:** Falls, Associated factors, Older adults, Environment hazards, Health status

## Abstract

**Introduction:**

Falls among older adults are a significant cause of disability, injury, and death worldwide. The high incidence of falls in older adults, combined with the increased susceptibility to injury of the older adult population, leads to severe global health issues. Further studies are needed to comprehensively evaluate the typical personal and environmental risk factors of falls in the Iranian elderly population. Future preventive strategies and intervention programs will be based on these findings. The study determined the risk factors associated with unintentional falls among a representative sample of older adults living in Tehran, the capital city of Iran.

**Methods:**

The study design was cross-sectional. The target population was men/women aged 65 years and over from the general population living in 22 different districts of Tehran who were selected by stratified random sampling. The researchers gathered the data using validated questionnaires and observations. The informed consent was obtained from all participants before starting the interview. Multivariate logistic regression analysis examined the association between falls occurring during the past 12 months with demographics/ socioeconomics and fall-related predictor factors.

**Results:**

The risk of falls was higher in women (47.0%) and those aged ≥ 75 years (44.1%). Older adults who were married had their fall risk reduced by 36.4% compared with other types of marital status. Older adults who were illiterate (48.1%), housewives (47.0%), and always had concerns about living expenses (53.9%) tended significantly to have a higher risk of falls. Moreover, participants who live with their family were less likely to fall than those who live alone (36.5% vs. 40.4%). Persons with safe homes were less likely to experience falls than persons with unsafe homes (30.9% vs. 41.4%). The logistic regression analysis showed that the female gender, being worried about living expenses, home safety, functional behavior, and function factors, were independently associated with the risk of falls during the past 12 months.

**Conclusions:**

Our findings revealed that a wide range of intrinsic and extrinsic risk factors contributed to injurious falls; based on the literature, some are preventable. The present data may be helpful as a starting point and guide future efforts for health providers and policymakers to allocate additional resources and develop proper falls prevention or intervention programs at the community level.

## Background

A longer life expectancy due to improved social and health care services, increased quality of life, and lower birth rates in the past decades have contributed to the growing proportion of older adults globally [1, 2]. Old age can be associated with limitations of personal abilities and postural instability, leading to dependence on others and a need for more health and social services. Fall accidents are among the most disabling harms at this age [3, 4]. Approximately 28–35% of older adults over 65 will experience falls at least once a year, and half fall recurrently [3, 5]. Falls among older adults result from many pathophysiological, aging processes, behavioral, balance and gait instability, pharmacological, ethnicity, intergenerational bonding and cultural practices, and environmental factors [6–8]. Falls are complex events that are caused by a combination of intrinsic impairments and disabilities, which are often compounded by a variety of extrinsic factors, such as environmental hazards [5, 9]. Primary risk factors for falls have been categorized into four main dimensions: biological, behavioral, environmental, and socioeconomic factors [5]. According to research, falls in older adults are multifactorial events involving several aspects, including internal (patient-related), external (environment hazards-related), and behavioral (individual activity-related) [4, 10, 11]. The number of risk factors in each individual increases the risk of falling [11, 12]. The meta-analysis study reported pain, weakness, gait problems, bone and joint diseases, dizziness, and age as risk factors for falling among older adults in the community [12–15]. In recent years, some studies have reported on the impact of risk factors such as dementia, bone and joint disease, and depression on falls of older adults in the community [16–21]. Another systematic review with meta-analysis highlighted that four fall risk factor domains, including balance and mobility, medication, psychological, and sensory and neuromuscular risk factors, were each associated with an increased risk of recurrent falls [15].

Falls among older adults cause disability, injury, and death worldwide [3, 22]. The high occurrence of falls in older adults, together with the high vulnerability of the elderly population to injury, leads to serious global health difficulties. In addition to the direct commercial burden of medical treatment for fall-related injuries, falls can generate a downward spiral that leads to reduced physical activity and an increased risk of chronic conditions and loss of functional independence, thus burdening the health care system. In short, falls can create enormous costs for healthcare systems, older adults, and their families [5].

Not only do falls cause enormous costs in high-income countries [23–26], but the increasing rate of fall-related injuries is progressively affecting the health and ability of older adults in Low- and Middle-Income Countries (LIMCs) to execute daily tasks.

So, falls are becoming a significant public health problem in LIMCs, where populations are aging rapidly [3]. Although Iran is known as one of the youngest countries in the world, statistical indexes of the population show that the aging process has started in Iran, and the Iranian population is shifting from young to old. The proportion of people ≥ 65 years, which 2003 was about 7%, will increase to 20–25% in 2030 [27]. In Iran, the epidemiological pattern of injuries needs to be clarified and documented in detail due to inadequate national information registration and conducting of studies on a local level using only small sample sizes [28, 29]. According to some population-based and systematic review studies, falls are the third cause of death from unintentional injuries [28] and the second most common cause of non-fatal injuries [28, 29]. These conditions emphasize the need for more assessments directed toward older adults to predict the predisposing factors of injury to plan appropriate prevention and intervention programs [30]. Numerous fall prevention measures are available globally, ranging from single to multicomponent interventions, mainly focusing on exercises, medication review, correction of hearing and vision impairment, and environmental modification only [7]. Very few studies have focused on socioeconomic, intergenerational harmony, and culturally driven fall prevention measures, which will prevent falls among older adults to a much greater extent. Because of the multi-factorial nature of falls risk factors, numerous studies have shown that interventions can effectively reduce falls in older adults by concurrently targeting several intrinsic and extrinsic risk factors [5].

For injuries of the same severity, older adults experience more disability, longer hospital stays, extended rehabilitation periods, a higher risk of subsequent dependency, and a higher risk of dying. The good news is that most risk factors are modifiable, and many falls and fall-related injuries are preventable [5, 7, 31]. There is compelling evidence that risk factors of falls can be influenced by implementing targeted intervention strategies designed to modify various intrinsic and extrinsic factors that increase the likelihood of falling [5, 32].

Given the multifactorial etiology of falls [12], the risk factors and prevalence of falls or fall-related injuries vary, including by the age of the target population, country, outcomes, and covariates measured. According to the literature, many fall-related injuries are preventable through managing the associated risk factors [5, 7]. Setting up a sound evidence base of adequate fall preventive strategies requires a better understanding of the characteristics of accidental falls among older adults. In addition, there may be different patterns for fall injuries in different communities [33], pointing to some potential culture-specific protective factors against these events [34]. Additional research on falls in older Iranians is necessary to evaluate the typical personal and environmental risk factors comprehensively. Future preventive strategies will be based on these data [35].

Considering the increasing number of older adults in Iran, the limited number of community-based studies on falls, and the need for assured data concerning risk factors of falls among this population, the present study was designed to provide data about risk factors of falls. Thus, this study aimed to determine the risk factors associated with unintentional falls among a representative sample of older adults in Tehran.

## Methods

### Definition of falls

Based on a frequently used definition, a “fall event” is defined as somebody unintentionally coming to rest on the ground, floor, or another lower level with or without loss of consciousness [3, 4].

### Characteristics of safe and unsafe homes

In the present study, safe homes are places without risks and potential environmental hazards that may cause injurious falls or even death to those living there. So, an unsafe home is one where there are environmental hazards, e.g., hazardous floor coverings, a small amount of clutter, furniture or cords in traffic ways, inadequate lighting at night, decreased contrasts between steps, lack of non-slip surface (mat) in the shower, unsafe garden paths, pets underfoot, absence of rails in the bathroom or stairs, unsafe stairs, and a need for a ramp.

### Study design and setting

This population-based cross-sectional study was conducted between 2012 and 2013 to determine the risk factors associated with falls among a representative sample of men and women aged 65 years and older from the general population living in twenty-two different districts of Tehran who experienced at least one fall during the past 12 months.

### Sample size calculation and sampling methods

Assuming a confidence level of 95% and the prevalence of falls among men in the age group 65– 69 years of 13% [36] with a sample precision of 2.6%, we considered a sample size of 653 individuals sufficient to have a representative cross-sectional sample of community-dwelling people aged 65 years and older living in Tehran [37]. Detailed information about sample size calculation, sampling methods, and inclusion and exclusion criteria has been shown elsewhere [38].

### Measures

Data regarding demography and socioeconomic variables, fall-related predictor factors, and fall occurrence were gathered using validated questionnaires and observations. The “Falls Risk for Older People – Community Setting (FROP-Com)” questionnaire was used to determine predictor factors related to falls. The FROP-Com is a validated tool used in the community that demonstrated good reliability and a moderate capacity to predict falls and assess risk factors [39].

### Independent variables and domains

In the present study, the research team examined three domains of independent variables:

Domain 1: demographic, socioeconomic and environmental variables: sex (male, female), age category (65–74, ≥ 75), marital status (single: never married/divorced/separated, married, died spouse), an education level (illiterate, primary school, secondary school, college or university), main work (retired, self-employed, housewife, and other), living arrangements (alone, family: spouse/wife and children, other), sufficient income (no, less than required, yes, enough to meet needs), housing situation (own, rental, different), worries about living expenses (always concerned, less concerned), environmental hazards (was there anything in the area around where the most recent fall occurred that contributed to the fall: no environmental hazards, involvement of environmental hazards) and home safety (is your home safe? no, yes);

Domain 2: health variables: having chronic condition/s (yes, no), number of chronic condition/s affecting balance and mobility? (none, 1–2 apply, 3–4 apply, five or more apply), number of prescription medications (no medication, 1–2 medications, three or more medications), number of medication types (such as sedative, antidepressant, and neuroleptics) (none, 1–2 types, three or more types), hearing problem (no, yes), vision problem (no, yes), sensory loss (uncorrected vision deficit (no, yes), uncorrected somatosensory deficit (no, yes)), feet problems (no, yes), cognitive status: ATMS score (9–10, 7–8, 5–6, 4 or less), continence (yes, no), balance (does the individual, upon observation of walking and turning, appear unsteady or at risk of losing their balance?: no unsteadiness observed, yes, minimally unsteady, yes, moderately unsteady, yes, severely unsteady), self-health status (good, acceptable, bad) and quality of life assessment (good, acceptable, bad);

Domain 3: functional behavior and performance variables: exercise except walking (yes, no), functional behavior (observed behaviors in Activities of Daily Living (ADL), and mobility indicate: awareness of current abilities, risk-taking behavior, under-estimates abilities, over-estimates abilities), function: (required assistance doing ADL/Instrumental Activities of Daily Living (IADL) before falls: completely independent, need supervision, some assistance required, completely dependent), gait:(walk safely around their home/ community: independent, no gait aid needed, independent with a gait aid, safe with supervision/ assistance, unsafe) and physical activity level (very active, moderately active, not very active, inactive).

### Dependent variables

The dependent variable was the occurrence of falls during the past 12 months (Yes/No). The data was collected through face-to-face interviews and observations with previously trained interviewers at the participants’ homes.

### Statistical analyses

The data were analyzed using Statistical Product and Service Solutions (SPSS) software version 23 (IBM Corporation). Analyses were carried out to address demographics/socioeconomics and the occurrence of falls, using univariate analyses to assess significant associations between falls and independent variables and examine each variable’s frequency distribution and summary measures. The Chi-square and Fisher’s exact tests were used to evaluate the relationship between categorical variables (all three domains of independent variables) and fall occurrence. The level of significance was set at *p* < 0.05.

Finally, a multivariate logistic regression analysis (Method = Backward Stepwise (Likelihood Ratio)) was conducted with the enter method, initially considering all variables with *p* values ≤ 0.10 to examine the independent association between dichotomous/categorical covariates (e.g., demographics/ socioeconomics, predictor factors) and the odds of having experienced a fall in the past 12 months among all participants [40]. Regression results were presented as odds ratios (ORs) and 95% confidence intervals (CIs).

## Results

### Characteristics of the participants

A total of 653 individuals participated in the study, with a mean age of 74.1 years (SD = 6.37). Most of the participants were women (50.8%), married (67.7%), and lived in their apartment or house (87.6%). Almost 83% of the participants lived with their family, 15.2% lived alone, 40.8% had primary school education, 50.9% were housewives, 65% lived with their spouse/spouse and children, 44% were worried about their living expenses, and 59.6% did not have sufficient income.

Of the 653 participants, 259 (39.7%) reported at least one fall in the previous 12 months, 166 (25.4%) fell once and 93 (14.2%) fell twice or more. Of the 259 fallers, 81.9% reported injuries, of which 52.9% complained of minor injuries not requiring medical attention, 17.8% of moderate injuries (e.g., large bruises), and 11.2% of severe injuries (e.g., fractures) requiring medical attention.

### Occurrence of falls by demographic, socioeconomic, and environmental factors

As shown in Table 1, the risk of falls was higher in women (47.0%), those aged ≥ 75 years (44.1%), illiterate (48.1%), housewives (47.0%), and always had concerns about living expenses (53.9%). Older adults who were married had their fall risk reduced by 36.4% compared with other types of marital status. Moreover, participants who live with their family (spouse/wife and children) were less likely to fall than those who live alone (36.5% vs. 40.4%).

**Table 1:** Demographic, socioeconomic and environmental variables by fall occurrence during the past 12 months (n = 653)

Variables	No falls			Falls				
	n (%)^1^			n (%)^1^		*Test* ^*a,b*^		
***Sex***						(χ^2^(1) = 15.14, *p* < 0.001)^a^		
Male	218 (67.9)			103 (32.1)				
Female	176 (53.0)			156 (47.0)				
***Age category (years)***						(χ^2^(1) = 4.11, *p* = 0.043)^a^		
65–74	237 (63.7)			135 (36.3)				
75 +	157 (55.9)			124 (44.1)				
***Marital status***						(χ^2^(2) = 6.01, *p* = 0.050)^a^		
Single; (never married, Separated / Divorced)	5 (55.6)			4 (44.4)				
Married	281 (63.6)			161 (36.4)				
Died Spouse	108 (53.5)			94 (46.5)				
***Education level***						(χ^2^(3) = 11.32, *p* = 0.010)^a^		
Illiterate	96 (51.9)			89 (48.1)				
Primary school	162 (60.9)			104 (39.1)				
Secondary school	95 (64.6)			52 (35.4)				
University or college	41 (75.5)			14 (25.5)				
***Main work***						(χ^2^(3) = 18.19, *p* = 0.000)^a^		
Retired	155 (67.4)			75 (32.6)				
Self-employed	46 (75.4)			15 (24.6)				
Housewife	176 (53.0)			156 (47.0)				
Other^d^	17 (56.7)			13 (43.3)				
***Living arrangements***						(χ^2^(2) = 7.17, *p* = 0.028)^a^		
Alone	59 (59.6)			40 (40.4)				
Family (spouse/wife and children)	270 (63.5)			155 (36.5)				
Other^e^	65 (50.4)			64 (49.6)				
***Sufficient income***						(NS)^b^		
No, less than required	233 (59.9)			156 (40.1)				
Yes, enough to meet needs	161 (61.0)			103 (39.0)				
***Housing situation***						(NS)^b^		
Own	350 (61.2)			222 (38.8)				
Rental	29 (55.8)			23 (44.2)				
Other^f^	15 (51.7)			14 (48.3)				
***Worries about living expenses***						(χ^2^(1) = 7.17, *p* = 0.007)^a^		
Always concerned	35 (46.1)			41 (53.9)				
Less concerned	358 (62.0)			219 (39.8)				
***Environment***: *1. Involvement of Environmental Hazards for falling*						(χ^2^(2) = 653.0, *p* < 0.001)^a^		
No fallers	394 (100.0)			0.0 (0.0)				
No environmental hazards	0.0 (0.0)			99 (38.2)^2^				
Involvement of hazards	0.0 (0.0)			160 (61.8)^2^				
***Environment***: *2. Home Safety*						(χ^2^(1) = 3.69, *p* = 0.055)^c^		
Unsafe home	319 (58.6)			225 (41.4)				
Safe home	65 (69.1)			29 (30.9)				

^1^=% within each variable; ^2^=% within fall occurrences; ^a^=chi-square; ^b^= NS, not significant; ^c^=Marginally significant, ^d^=e.g., worker; ^e^=e.g., friends; ^f^=e.g., homes belonging to relatives

Among those who experienced falls, the proportion of involvement in environmental hazards was more than those who reported no environmental hazards involvement (61.8% vs. 38.2%). Persons with safe homes were less likely to experience falls than persons with unsafe homes (30.9% vs. 41.4%).

### Occurrence of falls by health status factors

As shown in Table 2, the risk of falls was higher in older adults who had more than three types of chronic conditions (40.5%), used three or more prescription medications (48.4%), and had three or more types of medications (36.0%). Moreover, older adults were more likely to have fallen if they had hearing (47.1%) and vision problems (47.2%), sensory loss (uncorrected vision (52.3%) and somatosensory deficit (56.7%), feet problems, cognition problems, and urinary incontinence. The risk of falls was also higher in persons who had balance problems and were unsteady during walking and turning. Furthermore, older adults who reported poor self-rated health status and bad self-rated quality of life were more likely to fall.

**Table 2:** Health related variables by falls occurrence during the past 12 months (n = 653)

Variables	No falls			Falls				*Test* ^*a,b*^			
	n (%)^1^				n (%)^1^						
***Having chronic conditions***								(χ^2^(1) = 8.09, *p* = 0.004)^a^			
Yes	353 (58.7)				248 (41.3)						
No	41 (78.8)				11 (21.2)						
***Number of Medical Condition/s affected on movement and balance***								(χ^2^(3) = 37.17, *p* < 0.001)^a^			
None	107 (73.3)				39 (26.7)						
1– 2 types	207 (64.3)				115 (35.7)						
3– 4 types	62 (46.6)				71 (53.4)						
5 or more types	18 (34.6)				34 (65.4)						
***Number of prescription medications***								(χ^2^(2) = 19.28, *p* < 0.001)^a^			
No medication	74 (74.0)				26 (26.0)						
1– 2 medications	171 (64.8)				93 (35.2)						
3 or more medications	149 (51.6)				140 (48.4)						
***Number of medication types***								(χ^2^(2) = 24.02, *p* < 0.001)^a^			
None	187 (70.3)				79 (29.7)						
1– 2 types	183 (56.0)				144 (44.0)						
3 or more types	24 (40.0)				36 (60.0)						
***Hearing problem***								(χ^2^(1) = 4.82, *p* = 0.028)^a^			
No	311 (62.7)				185 (37.3)						
Yes	83 (52.9)				74 (47.1)						
***Vision problem***								(χ^2^(1) = 8.29, *p* = 0.004)^a^			
No	273 (64.4)				151 (35.6)						
Yes	121 (52.8)				108 (47.2)						
***Sensory loss***: *1. Uncorrected vision deficit*								(χ^2^(1) = 10.99, *p* = 0.001)^a^			
No	331 (63.5)				190 (36.5)						
Yes	63 (47.7)				69 (52.3)						
***Sensory loss***: *2. Uncorrected somatosensory deficit*								(χ^2^(1) = 26.59, *p* < 0.001)^a^			
No	323 (66.1)				166 (33.9)						
Yes	71 (43.3)				93 (56.7)						
***Foot problem***								(χ^2^(1) = 6.30, *p* = 0.012)^a^			
No	336 (62.6)				201 (37.4)						
Yes	58 (50.0)				58 (50.0)						
***Cognitive status***: *ATMS score*								(χ^2^(3) = 17.55, *p* = 0.001)^a^			
9–10	259 (66.1)				133 (33.9)						
7–8	88 (56.8)				67 (43.2)						
5–6	33 (44.6)				41 (55.4)						
4 or less	14 (43.8)				18 (56.3)						
***Continence***								(χ^2^(1) = 7.59, *p* = 0.006)^a^			
Yes	331 (62.9)				195 (37.1)						
No	63 (49.6)				64 (50.4)						
***Balance***: *Keeping balance walking and turning observation*								(χ^2^(3) = 45.10, *p* < 0.001)^a^			
No unsteadiness observed	290 (70.0)				124 (30.0)						
Yes, minimally unsteady	78 (44.3)				98 (55.7)						
Yes, moderately unsteady	18 (43.9)				23 (56.1)						
Yes, severely unsteady	8 (36.4)				14 (63.6)						
***Self-Health status***								(χ^2^(2) = 15.99, < 0.001)^a^			
Good	29 (69.0)				13 (31.0)						
Acceptable	143 (71.9)				56 (28.1)						
Low	217 (55.5)				174 (44.5)						
***Self-Quality of life assessment***								(χ^2^(2) = 10.43, *p* = 0.005)^a^			
Good	167 (65.7)				87 (34.3)						
Acceptable	190 (59.7)				128 (40.3)						
Low	35 (45.7)				34 (54.3)						

^a^=chi-square; ^b^= Fisher’s exact test; ^c^=NS, not significant; ^d^=e.g., worker; ^e^=e.g., friends; ^f^=e.g., homes belonging to relatives

### Occurrence of falls by functional behavior and performance factors

As shown in Table 3, the risk of falls was higher in older adults who did not exercise other than walking (42.4%) and persons whose observed behaviors in performing ADL and mobility indicated that they were underestimating their abilities (77.4%). Moreover, older adults who were entirely independent in doing ADL (35.9%) and IADL (33.7%) and could walk without gait aid and safely around their own home (36.6%) and community (34.5%) were less likely to fall. Notably, there was a gradient of protective effect from the risk of falls by physical activity level, with less risk for very active older adults and a higher risk for physically inactive persons (29.7% vs. 63.3%).

**Table 3:** Functional behavior and performance variables by falls occurrence during the past 12 months (n = 653)

Variables	No falls		Falls		*Test* ^*a,b*^
	n (%)^1^		n (%)^1^		
***Exercise except walking***					(χ^2^(1) = 8.79, *p* = 0.003)^a^
Yes	90 (72.0)		35 (28.0)		
No	304 (57.6)		224 (42.4)		
***Functional behavior***: *Observed behaviors in ADL and mobility*					(χ^2^(3) = 29.51, *p* < 0.001)^a^
Aware of current abilities	333 (64.8)		181 (35.2)		
Risk-taking behavior	53 (51.5)		50 (48.5)		
Under-estimates abilities	7 (22.6)		24 (77.4)		
Over-estimates abilities	1 (20.0)		4 (80.0)		
***Function***: *1. Required assistance doing ADL before falls*					(χ^2^(3) = 26.28, *p* < 0.001)^a^
Completely independent	341 (64.1)		191 (35.9)		
Need supervision	32 (58.2)		23 (41.8)		
Some assistance required	16 (29.6)		38 (70.4)		
Completely dependent	5 (41.7)		7 (58.3)		
***Function***: *2. Required assistance doing IADL before falls*					(χ^2^(3) = 17.64, *p* = 0.001)^a^
Completely independent	272 (66.3)		138 (33.7)		
Need supervision	21 (48.8)		22 (51.2)		
Some assistance required	85 (52.1)		78 (47.9)		
Completely dependent	16 (43.2)		21 (56.8)		
***Gait***: *1. Walk safely around their own home*					(χ^2^(3) = 15.06, *p* = 0.002)^a^
Independent, no gait aid needed	329 (63.4)		190 (36.6)		
Independent with a gait aid	54 (54.0)		46 (46.0)		
Safe with supervision/ assistance	9 (34.6)		17 (65.4)		
Unsafe	2 (25.0)		6 (75.0)		
***Gait***: *2. Walk safely in the community*					(χ^2^(3) = 29.86, *p* < 0.001)^a^
Independent, no gait aid needed	307 (65.5)		162 (34.5)		
Independent with a gait aid	65 (55.6)		52 (44.4)		
Safe with supervision/ assistance	18 (39.1)		28 (60.9)		
Unsafe	4 (19.0)		17 (81.0)		
***Physical activity level***					(χ^2^(3) = 21.08, *p* < 0.001)^a^
Very active	83 (70.3)		35 (29.7)		
Moderately active	197 (64.0)		111 (36.0)		
Not very active	96 (53.9)		82 (46.1)		
Inactive	18 (36.7)		31 (63.3)		

^1^=% of within each variable; a = chi-square

### Factors associated with falls

As shown in Table 4, gender, being worried about living expenses, home safety, functional behavior, and function factors were independently associated with the risk of falls during the past 12 months. Women were 1.76 times more likely to report a fall during the past 12 months than men were (OR = 1.76; 95% CI = [1.25–2.49]; *p* = 0.001). Persons less concerned with living expenses were more than 50% less likely to report a fall in the past 12 months than persons who were always concerned (OR = 0.48; 95% CI [0.29–0.81]; *p* = 0.006). People with safe home environments were about 40% less likely to report falls in the past 12 months than those with unsafe homes (OR = 0.61; 95% CI [0.37–1.02]; *p* = 0.059). Persons with under-estimates abilities/inappropriately fearful of activity were 4.51 times more likely to report a fall during the past 12 months than those aware of their current abilities (OR = 4.51; 95% CI [1.57–12.92]; *p* = 0.005).

Persons who required some assistance for personal care ADL were 2.33 times more likely to report a fall during the past 12 months than persons who were completely independent (OR = 2.33; 95% CI [1.11–4.91]; *p* = 0.026).

**Table 4:** Independent predictor factors for falls in men and women aged 65 and over (n = 653)

Independent Variables	*p*-value	OR^1^	(95% CI^2^)	
			*Lower*	*Upper*
***Sex***				
Men ^a^		1.00		
Female	0.001	1.76	1.25	2.49
***Concerns about living expenses***				
Always concerned with living expenses ^a^		1.00		
Less concerned with living expenses	0.006	0.48	0.29	0.81
***Home safety***				
No ^a^		1.00		
Yes	0.059	0.61	0.37	1.02
***Functional behavior***^***b***^: *Observed behaviors in ADL and Mobility*				
Aware of current abilities ^a^		1.00		
Risk-taking behavior	0.078	1.53	0.95	2.46
Under-estimates abilities	0.005	4.51	1.57	12.92
Over-estimates abilities	0.135	7.57	0.53	107.96
***Function***^***b***^: *Required assistance for personal care ADL before falls*				
Completely independent ^a^		1.00		
Need supervision	0.585	0.84	0.44	1.58
Some assistance required	0.026	2.33	1.11	4.91
Completely dependent	0.579	0.64	0.13	3.14

^1^ OR: Odds Ratio; ^2^ CI: confidence interval; ^a^ Reference group; ^b^ Categorical variables

The Cox and Snell’s R-square was 0.126, indicating that the logistic model explained 12.6% of the variance in the dependent variable. The Nagelkerke R squared was 0.171, showing a relationship of 17.1% between the predictor and the prediction.

## Discussion

### Overview of the results

This study aimed to determine the risk factors associated with unintentional falls among older adults. Firstly, we evaluated the relation between falling with each determinant in three domains. These domains were demographic, socioeconomic, and environmental factors, health status; and functional behavior and performance.

### Demographic, socioeconomic, and environmental factors

Considering demographic and socioeconomic factors, the fallers were more likely to be women in the age group of over 75, with lower education level, being housewives, had always concerned about living expenses, less likely to be married, live with their family (Spouse/Wife and children), have their own house and involved in environmental hazards were more likely experience falls.

In line with our results, other researchers have argued that as people age, they become more prone to falls [9]. After age 60 and aging, the prevalence of falls and the severity of the complications increase, as well as mortality among fallers in both sexes [9, 41]. Studies highlighted the effect of demographic factors, including old age (especially people over 75) and living alone [41]. Both men and women are at risk of falling, but older women are more at risk [3, 42–44]. A reason could be a weaker musculoskeletal system and osteoporosis in women, often associated with hormonal changes during menopause [45, 46]. Ghanbari et al. [47] reported a relationship between falls and being a woman, with a prevalence of falls at 6.45% in women compared to 2.25% in men [47]. On the other hand, Nabavi et al. [48] did not find differences in the frequency of falls between men and women, which may be due to the same conditions in their living environment.

By our results, Davoodi et al. [49] found that education had a significant relationship with falling. Additionally, the studies of Marashi et al. [50], Abbasi et al. [51], and Salarvand et al. [52] showed that falls are higher among illiterate older adults. Li et al. [53] found that the frequency of falls was inversely related to the educational levels of the fallers. Gill et al. [54] also observed that more years of education was a protective factor against falls among Australian community residents. This protection could be due to the greater awareness of older adults with higher education about fall risk factors and the advantage of their education, which helps them avoid situations that increase the odds of falls. However, Nabavi et al. [48] found no significant relationship between education and falling. The absence of such a relationship could be because most older adults in these two studies had a basic literacy level. Conversely, Nabavi et al. [48] found no significant relationship between education and falling, probably because most older adults in this study had a basic literacy level.

In line with our results, Marashi et al. [50] and Salarvand et al. [51] found that the highest rate of falls was in older adults without a spouse. Based on some previous studies, married persons have more access to resources regularly due to multiple incomes and a better economy that can improve their health status [55] and may prevent them from injurious falls. Thus, in the present study, social and economic support provided by a spouse and strong family connections among married elders might be associated with their more significant participation in social or leisure activities. These activities encourage healthier lifestyles that prevent them from falling. The direct relation between living environment hazards and the risk of falling is consistent with the literature review arguing that the hazards in a poorly designed and unsafe home and environment can increase the risk of falls [56, 57]. The extent of injury following a fall depends on patient-related and environmental factors [58]. The environmental factors that contribute to the risk of falls in older adults were recognized, including polished, wet, and slippery floors, slippery carpets in the home, wires or small objects on the floor, insufficient ambient light, no handles in the bathroom and toilet are among the environmental hazards [59, 60]. The absence of significant safety design features in the environment is one of the causes of falls among older adults. Thus, the improvement in home safety design using home fall-hazard interventions substantially reduces falls and prevents people from falling [61, 62].

### Health status factors

The older adults who experienced falls were more likely to have chronic medical conditions (more than three conditions), take multiple medications (more than three medications), and take a variety of medications (more than one type of medication). However, most fallers were more likely to have hearing and vision problems, sensory loss, and foot problems, having lower scores in cognitive status, but they were better in urinary continence. They were also more likely to have a balance problem, low self-rated health status, and self-rated quality of life.

In line with our findings, falling can be related to poor health and performance and is often associated with a serious underlying disease [63–66]. Multi-medication, cognitive, and sensory impairments have been reported as risk factors for falls in older adults [36, 67–69]. The higher prevalence of health problems and comorbidities in old age makes older adults prone to falls [36, 67]. Considering the older adult population growth in Iran, the prevalence of chronic diseases (CD) has also increased, and consequently, health needs in the population will increase [70, 71]. The high prevalence of CD is often associated with a higher probability of falls and death [72]. In the study of Jafarian Amiri et al. [73], the incidence of falls in older adults with a history of chronic disease was 5.4 times higher than in older adult who was not sick. Chronic conditions such as heart disease, hypertension, hypotension, diabetes, seizures, headaches and dizziness, bone and joint diseases, balance, gait disorders, and vision problems in an older adult can lead to disability and cause the occurrence of falls in the older adult [68, 69, 73].

Medications are usually a solvable risk factor in older adults. Older adults are prone to take multiple medications because of multi-morbidity. Older adults are often sensitive to some medications, affecting their balance or ability to think clearly [74]. Taking too many medications can lead to side effects such as confusion, increasing the prevalence of poisoning and injuries in older adults [75]. Older adults, especially when taking several drugs simultaneously, are more exposed to the risks of side effects and interactions of drugs [76]. Also, physiological changes following aging reduce older adults’ ability to metabolize and eliminate the side effects of drugs [68]. Other scenarios of intoxication in older adults include repeated doses, unintentional use of the wrong medication due to poor eyesight, medication of other family members, and eating what is not edible [74–76].

Some medications are well-known for side effects that increase a person’s risk of falling [74]. The more drugs the older adults take, the greater the chance that a combination of them will make a fall more likely to happen [74, 75]. According to our results, using four or more prescription medications is considered an established risk factor for falls in older adults [77]. The risk of hospitalization due to falls increased with polypharmacy [74, 75, 77]. Anti-hypertensive medications and medications that suppress the central nervous system (reduce alertness and cause slower reactions and movements) are among those most likely to contribute to falling [74, 75].

As in the present study, chronic illness, problems with balance, and weakness are health-related risks for falling [41, 45, 46]. Sensory loss and foot problems were common among fallers in this study. In agreement with our results, studies have shown that elders’ feet need special care because the risks for chronic foot problems such as arthritis, gout, and diabetes increase with age [78]. Chronic feet numbness is a common problem in people with diabetes for a long time and can affect how older adults stay on their feet and keep their balance [77].

Age-related sensory loss is a serious problem. A previous study by Pinto’s team [79] showed that dysfunction in olfactory predicted mortality better than a diagnosis of cancer or heart failure. Researchers found that older adults had more sensory deficits, with significant differences in hearing, vision, and smell [79]. Age-related sensory loss is an understudied issue [78]. Among older adults, sensory impairment is common. It is often a precursor of serious health problems such as cognitive decline or falls and subtler ones like burns caused by decreasing sense of touch [78, 80]. The typical process of underlying factors in sensory impairment could include neurodegeneration, environmental insults, or an underlying genetic susceptibility contributing to age-related sensory loss [77–80].

#### Functional behavior and performance factors

Concerning functional behavior and performance, the risk factors for falls included: less likely to do exercise except walking, more likely to under-estimate or over-estimate abilities, the fallers were less likely to be independent in ADL, IADL in walking, and needed no gait aid when walking safely around their own home and in the community. Fallers were also more likely to be inactive.

It is well-documented that falls are the leading cause of injuries and emergency ward visits in people over 65 [41, 45]. Many serious injuries, including concussions, soft tissue injuries, fractures, and bone dislocations, occur in people with falls [46, 47]. Pelvic fracture is among the most common causes of hospitalization in older adults following falls, which can increase mortality and fall-related injury and reduce the quality of life [49, 81]. Falls usually do not cause fractures or death [82]. However, many older adults experienced a fear of recurring falls after a fall, resulting in decreased activity and increased inactivity and dependence [83].

In agreement with the present results, older adults are more likely to fall due to muscle weakness and balance problems [84]. The age-related comorbidities include osteoporosis, decreased physiological functions such as reduced operating time and reactions, and slowness of reflexes and responses [85]. These make even a mild fall in older adults potentially dangerous and problematic [84, 85].

### Logistic regression analysis

A Logistic Regression model weighed the overall effects of all risk factors. Considering the odds ratios, the present study concluded that being a woman, underestimating their abilities, and needing assistance for ADL were risk factors for falls, whereas being less concerned with living expenses and having a safe home were protective factors.

Our model partially confirms the results of other studies [9, 41, 59, 86]. They showed that older adults are more at risk of falling due to a combination of risk factors. Being a woman, dependent on doing ADL, having a lower income, and having a less safe environment [36, 67] have been reported as the risk factors for falling. This model also confirms that falling is a “multi-factor” event, which means several factors usually lead to the fall or fall risk in aging [67, 87].

Compared to men, women have less muscle mass and a weaker musculoskeletal system [88, 89]. Women also are shown to have lower SES [53, 64, 86], do less regular exercise, and have more disability [90, 91], which can lead to falls. Being dependent on others or assistance devices and disability are linked to falls [92].

Our findings align with studies showing an association between lower income and an increased risk of falling [63, 86]. Older adults, especially women with unreliable and insufficient income, face an increased risk of falls. This increased risk could be because lower income is associated with a poor living environment, poor health behaviors, and barriers to health care services, which affect the individual’s health status and increase the risk of falls [41, 63, 93–95].

Despite extensive research on individual risk factors in older adults, the possibility of predicting who will fall and who will not is still relatively low [85, 92], possibly because risk factors interact rather than have an independent effect. Specific patterns of risk factors associated with falls have shown that combining risk factors results in the best prediction of future fall risk [36, 58, 66, 67].

### Highlights of the present study, limitations, and suggestions

The generalizability of the present study is confirmed. This study evaluated the relationship between falling with a wide variety of variables, including demographic, socioeconomic, environmental, health status, and functional behavior and performance in a representative sample of community-dwelling older adults living in Tehran that this city, in turn, included a representative sample of Iran [96]. Most of the conducted studies in Iran had a small selection of older adults living in a small community, in elderly care centers, or under hospital care [25, 48, 52]. Getting informed regarding the effect of different risk factors can help to develop targeted and comprehensive intervention/prevention strategies to reduce falls among older adults.

However, this study provides only cross-sectional data. More robust designs would help to evaluate causal relations (e.g., a longitudinal study with repeated measures). In addition, the data collected solely depended on the participants’ subjective assessments, and the data included no objective criteria to corroborate their responses. Also, age-related cognitive deficits may have affected the reporting of some interviewees, although most participants were in good cognitive status. Another limitation of the study was the exclusion of subjects who were bedridden or in wheelchairs and unable to walk or consent to participate. The exclusion of such individuals with physical frailty who may be at increased risk of falls may lead to an underestimation of the effects of the risk factors.

To avoid bias in recalling questions such as the history of the falling, we asked about a specific and well-known event about a year ago and the interview date to help participants remember their crash events in the past 12 months. To avoid biasing information about variables such as age and gender, we used their national ID card. Variables such as fall history and level of education are prone to information bias, which is one of the design limitations. We tried to avoid such biases by interviewing an informed person who lived with each participant. Despite these limitations, our study confirms previous findings and may offer new insights into falls and related factors among older adults.

Falling and prevention of related injuries need multidisciplinary management [97]. One of the critical aspects is knowing the risk factors of falls in older adults, which will help them choose appropriate prevention strategies to help themselves recognize and avoid risky situations. Learning about the effects of medical conditions and types of medications would help older adults ask doctors to review their situation and find out how to eliminate medication to help prevent falls in the future. On the other hand, learning the risk factors of falls in older adults means that the care systems understand why the older adults fall and take steps to help them. However, personalization is essential in preventing older adults from falling [97]. Once we identify the factors that specifically cause the older person to fall and focus on solving them, we can personalize our approach to preventing these particular older adults from falling.

## Conclusions

In the present study of men and women aged 65 or over, we evaluated the relationship between various factors and the risk of incident falls. Being a woman, at the age group of over 75, lower education level, being a housewife, always having concerns about living expenses and not being married, not living with their family (spouse/wife and children), not having their own house, facing environmental hazards, having chronic medical conditions (more than three conditions), take multiple medications (more than three medications), and take a variety of medications (more than one type of medication). Having problems in hearing, vision, foot, cognition, and balance; having sensory loss; poor self-rated health status and self-rated quality of life; doing less exercise; having under-estimates or over-estimates abilities; not being independent in ADL, IADL in walking, and needing gait aid when walking safely around their own home and in the community, and being inactive. Checking the overall relations, the logistic regression model confirmed that being women, under-estimating their abilities, and needing some assistance for doing ADL (required assistance for personal care ADL before falls) were risk factors for falls, whereas less concerned about living expenses and having a safe home were protective factors.

Our findings revealed that complex interaction among a wide range of intrinsic and extrinsic risk factors, including biological, socioeconomic, behavioral, and environmental factors, contribute to falls resulting in injuries. Most risk factors are modifiable, and most falls can be prevented among older adults. In Iran, appropriate multicomponent fall prevention and intervention is needed to prevent older adults from falling and falling-related. The present data may be helpful as a guide to future efforts for health providers and policymakers to allocate additional resources and develop proper falls prevention/intervention programs at the community level.

### Implications

Our study accentuates the need for a broad assessment of older adults, including physical and psychological health, social and financial resources, education, environment, physical activity level, functional behavior, and performance. Prevention strategies and treatment include correcting the underlying causes of the fall, treating or controlling the disease, improving their health and socioeconomic status, restoring their health-related behavior, and returning the older adults to an acceptable level of activity and functional performance.

## Data Availability

The datasets generated and analyzed during the current study are not publicly available due to privacy concerns. Still, they are available from the corresponding author upon reasonable request and with permission from the Sina Trauma and Surgery Research Center (STSRC).

## Abbreviations

LIMCs: Low and Middle-Income Countries
FROP-Com: Falls Risk for Older People-Community Setting
ADL: Activities of Daily Living
IADL: Instrumental Activities of Daily Living
SPSS: Statistical Product and Service Solutions
TUMS: Tehran University of Medical Sciences
STSRC: Sina Trauma and Surgery Research Center
ORs: Odds Ratios
CIs: Confidence Intervals
NS: Not Significant
